# American Dental Association

**Published:** 1886-03

**Authors:** A. M. Dudley

**Affiliations:** Salem, Mass.


### The American Dental Association.

The Union Pacific Railway Company and connecting lines have agreed to sell members of the American Dental Association and delegates to the same and all dentists desiring to attend the meeting of the Association, and their families, round trip tickets to San Francisco and return at the very low rates to be given to the Grand Army of the Republic, provided the Association will hold its next annual meeting in San Francisco the last week in July of the coming summer. The other trans-continental lines will probably soon give us the same terms.

---

register, and shall have taken out their tickets of admission, and such other scientific men as the Executive Committee of the Congress may see fit to admit."

"Dentists not holding the medical degree are included in the provision for admitting "other scientific men," and it is *now* understood that such gentlemen as the Officers and Council of the Section recommended will be invited to full membership in the Congress. In this way the Section is made responsible for the respectability and professional attainments of the membership.

A. W. H.

The round trip rate will be $50 from Council Bluffs, Ia., Omaha, Neb., Leavenworth, Kan., St. Joseph, or Kansas City, Mo., to San Francisco and return. About $60 from Chicago, Cincinnati, and St. Louis, and $75 from Atlantic Coast. No extra charge for returning by one of the other Pacific lines to its eastern terminus if desired and arranged before the journey is begun. If exchange of ticket is made after arriving in San Francisco, $10 additional is charged.

Free baggage allowance, 150 pounds per ticket.

Round trip rates from San Francisco to interior and coast points will be exceedingly low.

Return tickets via Portland and the Oregon Short Line or the Northern Pacific will be sold, at Missouri River, the additional charge being only $12.50, for steamer passage, San Francisco to Portland, provided route is selected when ticket is purchased.

Special trains will be given us with privilege of stopping at desirable points such as Denver, Salt Lake City, etc., and the schedule so arranged as to cross the Rocky and Sierra Nevada mountains by daylight. Everything will be done to make the trip a pleasant one for us. Tickets will be from July 1st for 30 days going and for 85 days returning. The California State Odontological Society have taken decided and favorable action in favor of our going. It seems to be an opportunity to hold our meeting on the Pacific Coast, which we should not lose. Copies of the U. P. Tourist Book had by writing to any office of Co. All members of the American Dental Association and all dentists who would be favorable to the meeting of the Association being held as above will please send their names to

A. M. Dudley, Salem, Mass.

